
JOURNAL INFORMATION
==============================
NLM Title Abbreviation: Med Chir Rev
NLM Journal ID: 0034617

The Medico-Chirurgical Review

Publisher: Biomedical Journal Digitization Project

ARTICLE INFORMATION
==============================
PMCID: PMC4966263
PMID: 29917633
Article version: 1
Subjects: Reviews, Diseases Produced by Malaria

Memoir on Visceral Neuralgia

Print publication date: 1828 Oct 1
Volume: 9
Issue: 18
First page: 333
Last page: 336
1828 Jul
License: This work is free of known copyright restrictions. For more information, please see PMC Back Issue Digitization.
License URL: https://creativecommons.org/publicdomain/mark/1.0/

==============================

doubt not that, while some attacks have been less destructive than others, we should, in our general deductions, study the character of the epidemic, in estimating the success of its treatment.

In a paper of such circumscribed limits, it was impossible to introduce every point and discuss every question, which attaches to a subject of so much practical interest as Hospital Gangrene; many particulars have been, consequently, overlooked, and some have been imperfectly noticed; but the topics, which the author has considered it proper to enlarge upon, have received justice, and, in the course of our observations, we have occasionally endeavoured to supply the blanks with important extracts and original remarks.

---

### VI.

### *Memoir on Visceral Neuralgia.* By P. JOLLY, M. D.

MUCH has been written on that class of painful maladies, the neuralgiæ of the nerves of relation—and, under the class *neuroses*, many diseases, or rather symptoms, connected with or dependent on visceral neuralgia, have been accurately described, as for example, angina pectoris, spasmodic asthma, hooping-cough, &c. Few or no investigations, however, have been made into the state of the visceral nerves themselves, on which the disorders of function in these viscera, so often depend. The general sentiment, indeed, appears to be that, in the *neuroses*, there is a total absence of appreciable change of structure, or, in other words, of physical lesion, in the parts affected. Our author thinks, on the contrary, that there are very few of the neuroses, whether external or internal, in which some lesion might not be detected either in one of the principal nerves, their divisions, their anastomoses, or in one of the great nervous centres. The researches of Bichat, Beclard, Swan, and others, on the local affections of nerves, justifies him in this opinion, as far as the nerves of relation are concerned. These researches have reduced the number of vague terms in medicine, and augmented the number of those which have some anatomical and physiological meaning. But we have not been so successful in our researches respecting the visceral or ganglionic nerves. All there is darkness; and we have hardly ventured to apply the term *neuralgia*—probably because the idea of great pain is associated with the word; whereas, the splanchnic nerves, having specific kinds of sensibility, may suffer severely in their own way, and greatly disorder the functions of the organs on which they are distributed, without any sensible pain, in the common acceptation of the word.

M. Jolly employs the term NEURALGIA, then, to designate every lesion, physical or vital, direct or indirect, of a nerve, unaccompanied by external signs of inflammation—assuming, for the most part, a periodical character—and disturbing, more or less, the functions of the corresponding viscera. He reserves the term "NEUROSES," on the other hand, for all those nervous affections where no local cause can be traced in the nerve, but merely disorder of function in the organ. Our author properly observes that, between the cerebro-spinal nerves (the nerves of relation with the outward world) and the ganglionic nerves arising from the solar plexus, there is an intermediate class (the phrenic and eighth pair) which partake of the characters and functions of both the others—and whose disorders are modified accordingly. It is with this class of nerves that our author commences his history of the neuralgiæ of the nutritive life—or the function of digestion and assimilation.

### PHRENIC NEURALGIÆ.

*Clinical Characters.* The phrenic neuralgiæ, like most nervous disorders, are generally intermittent. They manifest themselves by more or less of pain, accompanied by a sense of constriction about the epigastrium or in the back— by hiccup, often attended with eructations, vomiting, and other symptoms denoting a spasmodic affection of the diaphragm. They may also give rise to disorder, more or less marked, of the respiratory apparatus, and thus simulate all the phenomena of asthma. They are usually unattended with pyrexia; but, in some cases, they are accompanied by all the general symptoms of intermittent fever, simple or malignant.

### EXAMPLES.

*Case* 1. In the 5th volume of the Medical Journal of Vandermonde, M. Hazou has published a case of painful intermittent hiccup, which illustrates this subject. A lady, 30 years of age, received some melancholy news while menstruating, and by which the catamenial discharge was stopped. On this suppression, there supervened a most distressing hiccup, which lasted with great violence for 36 hours. There was then an intermission of 24 hours, when the hiccup returned with the same degree of intensity, and lasted the same number of hours. The complaint went on in this manner, the periods of attack and intermission as above, for several weeks, by which the patient was nearly brought to the grave. She was cured at last, by a course of strong purgative medicine.

*Case* 2. This case is published by M. Bigot, in the 92d number of the "CLINIQUE DES HÔPITAUX." Madeline Rabouin, aged 24 years, had been subject to occasional attacks of hiccup for five years, which generally lasted a few hours, and then ceased. During the last six months, the hiccup has seldom appeared, but it has been superseded by attacks of aphonia so complete as to render her incapable, for the time, of uttering a single sound. M. Bigot was consulted, and found that this young woman was not regular in her catamenia, and that she complained of pain in the back part of her head. She was directed to apply 15 leeches to the anus, and to encourage the discharge. By this remedy the aphonia was removed; but it was replaced by the periodical attacks of hiccup, which were infinitely more distressing than the aphonia. A tartar-emetic plaster was applied to the epigastrium, which brought out a plentiful crop of pustules, and these were kept in a state of irritation and discharge by fresh applications of the antimonial. This put an end to the singultus for 12 days, when it returned. A second plaster completely dissipated the complaint.

*Case* 3. (HOSPITAL PRACTICE.) Miss Mason, aged 27 years, of delicate constitution, and of great susceptibility of nerve, had been subject to indigestion since the age of 20, but was regular in her menstrual evacuations. Seven months prior to the date of report, she was, without any apparent cause, seized with a rigor, which lasted several hours, accompanied by loud and quickly repeated discharges of flatus from the stomach. These eructations continued, with more or less intensity from that period, the patient being seldom more than an hour without them. In this state she presented herself to M. Dupuytren, at the Hôtel Dieu, under a severe paroxysm, the head being bent forward on the chest, while the sterno-cleido, pectoral, and several other muscles, as well as the diaphragm, were constantly agitated by convulsive movements. The eructations were unattended with pain; but when the paroxysms lasted any considerable time, she appeared to be threatened with suffocation. She was greatly emaciated, but preserved her appetite, and had some sleep at night. M.

Dupuytren resorted at once to a powerful remedy—the actual cautery. Two irons, of two inches in diameter, and of a white heat, were held successively, close to the pit of the stomach, till the integuments became of a deep red colour. The cries of the patient, who was firmly held by assistants, were dreadful, and she sunk, at last, into a kind of stupor, and all spasmodic action ceased. On recovering, she was conveyed home, and, in the evening, she was at her usual occupations, and in good spirits. For some days after this, she had but very slight attacks, and when the report closed she was nearly well.

*Case* 4. (HÔTEL DIEU.—M. DUPUYTREN.) This was a female also, of irregular menstruation, who had been harrassed for two years, with a most distressing singultus, accompanied by spasmodic action of the pectoral and cervical muscles. M. Dupuytren employed the actual cautery in the same manner as in the preceding case. The skin was disorganized, but the singultus was stopped as by a charm. Fifteen days afterwards she returned to the hospital, and reported that the hiccup was now so mild that it caused her very little inconvenience.

Several authors have related cases of intermittent hiccup, accompanied with pain, more or less acute, in those organs, under the influence of the phrenic nerve, and which only gave way to large doses of the cinchona. These phrenic neuralgiæ, like all others of that class, may be accompanied by fever—and are then, in fact, intermittents. We have little doubt that they arise from the same causes that produce agues.

*Anatomical Characters.* These are very little known, as the subject has not been prosecuted to any extent. Our author has little doubt that a physical change in the nerve, or its neurilema, (probably of an inflammatory character) takes place. We find an instance recorded by the elder Berard, in the case of an individual who had presented all the symptoms of asthma. On dissection, no other physical change could be discovered than a small tubercle, the size of a pea, of a black colour, and extremely hard, which had completely interrupted the phrenic nerve on one side. It is certain, however, that neuralgia exists entirely independent of local lesion, and which appears to result from, and to be kept up by a vicious habit of the vital powers or properties of the nerve itself. Such would appear to be the case in the two instances related by M. Bigot, from the hospital practice of Dupuytren, and which would, probably, have given way to bark or arsenic, as well as to the actual cautery. Still it must be confessed, that the nerves have been but little examined in the neuroses and neuralgiæ, and it is, therefore, to be hoped, that future researches into the intimate texture of parts, will detect many lesions now unknown. Till then we must trust to external symptoms.

PNEUMO-GASTRIC NEURALGIÆ.

*Clinical Characters.* These neuralgiæ are infinitely more common than is suspected. It is to these, in fact, that we are to attribute the greater number of periodical or spasmodic asthmas, dyspnœas, periodical coughs, hooping coughs, and what have been called "nervous vomitings," by different writers. It is in this class we must place the "tussis suffocativus," quotidian, tertian, &c. of Galleazzi, Ridley, Home, Stork, and others. In this class must be located the periodical vomitings with epigastric pain, cited by Heister, and many other authors.

Those hemicraniæ that are so distressing and obstinate are probably referrible to this class of neuralgiæ, although *sympathetic* in respect to the immediate nervous seat of the pain. We shall select and condense a few cases illustrative of this class of visceral neuralgiæ.

*Case* 5. (M. DUMERIL—MAISON ROYALE.) A female cook, aged about

30 years, had been subject, for several years, to hemicraniæ, which attacked her many times in the month. They were ushered in by chilliness, succeeded by re-action, agitation, sense of strangulation, and, lastly, by sickness and vomiting. She was cured by anodynes and evacuants, followed by tonics.

*Case* 6.—(*Same Establishment.*) A female, aged 39 years, had been harrassed for some time with paroxysms of cough resembling hooping-cough. It generally came on every evening, especially after eating. To the paroxysms of coughing was added vomiting, with dreadful straining and convulsive agitation. She had been treated by leechings, purgings, diluents, and other means, when having entered the Maison Royale, M. Dumeril wisely considered the case as one of nervous periodical affection, and soon cured her by anodynes and tonics.

*Case* 7.—In a former number of the Bibliotheque Medicale, M. Jolly had published a case which shewed the difficulty of diagnosis, while it tends to corroborate the opinion of M. Fodera, that asthma is often dependent on a certain morbid condition of the brain and nerves. An individual had experienced, during several years, a train of anomalous symptoms in the vascular and respiratory apparatus, which led his physicians to suspect serious organic disease of the chest. On the least exertion of body or emotion of mind, he was threatened with suffocation, had violent palpitation of the heart, irregularity of the pulse, indigestion, apparent enlargement and pain in the liver. Bleeding and starvation did not mitigate the disease, while it seemed to accelerate general dropsy, of which he died. The most accurate examination after death, did not detect any deviation from healthy structure in the chest; and the only morbid anatomy that was found, consisted in a softening and vascularity about the corpora olivaria, near the origin of the par vagum.

*Anatomical Characters.* These, like the other neuralgiæ, may leave no cognizable trace of their existence in the nerves affected. This, indeed, is the case ninety-nine times in the hundred. Pathological anatomy, however, is not without examples of physical changes in the pneumo-gastric nerves, corresponding with the symptoms during life. Thus Autenrieth found the par vagum and phrenic inflamed in individuals who had died of spasmodic cough, resisting every kind of treatment. M. Breschet discovered the pneumo-gastric nerves of a yellow colour in people who had died of whooping-cough. Andral has related a remarkable case of asthma where the par vagum was altered in structure. Gendrin, Cruvelhier, and others, have also reported analogous cases.

It is to be remembered, however, that physical lesions in the nerves are rare when compared with disordered function, and this will apply to all other textures of the body as well as to the nerves. We are bound, therefore, to conclude that the physical change is one that takes place as a consequence, in general, of the functional disorder, though that consequence must, in its turn, prove a cause or an exasperation of the disorder.

But what we would wish particularly to impress on the minds of our younger brethren is the utility of attending to these visceral neuralgiæ, and especially to the periodical forms which they assume, leading us to trace them to the same general causes which produce the tribe of agues and remittent complaints—namely, miasmal emanations from the earth. It is on this supposition, that we can comprehend the alleviation which a trifling change of place and air will frequently produce in this class of complaints, while it explains the reason why some people cannot maintain a day's good health in certain localities. An attention to the medical topography of the place may often lead to a knowledge of the cause—and a knowledge of the cause is the surest key not only to prevention but to cure.
